# Dual RANSAC with Rescue Midpoint Multi-Trend Vanishing Point Detection

**DOI:** 10.3390/jimaging12040172

**Published:** 2026-04-16

**Authors:** Nada Said, Bilal Nakhal, Ali El-Zaart, Lama Affara

**Affiliations:** Department of Mathematics and Computer Science, Faculty of Science, Beirut Arab University, Riad El Solh, P.O. Box 11-5020, Beirut 11072809, Lebanon; b.nakhal@bau.edu.lb (B.N.); elzaart@bau.edu.lb (A.E.-Z.)

**Keywords:** vanishing point detection, RANSAC, multi-trend analysis, distance prior, line segment detector, robust estimation

## Abstract

Vanishing point detection is a fundamental step in computer vision that allows 3D scene understanding and autonomous navigation. Classical techniques have significant challenges when trying to understand scenes that are heavily cluttered and images containing multiple perspective cues, leading to poor or unreliable vanishing point determination. We present a Dual RANSAC with Rescue Midpoint-based Multi-Trend Vanishing Point Detection framework, which targets the simultaneous detection and fine-tuning of multiple, globally consistent vanishing points. The proposed framework introduces a novel Midpoint-based Multi-Trend Random Sample Consensus formulation that operates on line segment midpoints to infer dominant directional groups, thereby eliminating noisy or unstable midpoints and stabilizing subsequent vanishing point inference. The main novelty lies in using line segment midpoints to model the orientation variation as a linear regression in the midpoint–orientation space, which helps reduce sensitivity to endpoint instability. Candidate vanishing points are prioritized through inlier-based confidence ranking and subsequently optimized via an MSAC-based arbiter to resolve hypothesis conflicts and minimize geometric error. We evaluate our work against state-of-the-art techniques such as J-Linkage and Conditional Sample Consensus, over two of the current challenging public datasets that comprise the York Urban Dataset and the Toulouse Vanishing Point Dataset. The results show that the proposed framework achieves a recall of up to 95% and an image success rate of almost 84%, outperforming both J-Linkage and Conditional Sample Consensus, especially under tighter angular thresholds. This demonstrates the ability of the proposed framework to provide enhanced stability and localization accuracy.

## 1. Introduction

Detecting a vanishing point (VP) is a significant process in many computer vision tasks, and is essential for creating a 3D structure from a 2D image of a scene. In 3D space, parallel lines in a scene converge to one point in a 2D image due to perspective projection. Each of these points is defined as a vanishing point [1]. VPs are essential in 3D geometry-based tasks like scene reconstruction, camera calibration, layout reconstruction, and autonomous driving. Typically, in a 3D scene, several dominant directional cues are present (mostly horizontal, vertical, and oblique); hence, resolving VPs in 3D scenes is important for preserving the geometry of a scene.

Many works have focused solely on geometric-based approaches to VP detection. Due to their effectiveness, many geometric approaches [2] remain in practice. Hough-type voting algorithms are able to identify dominant directions [3], as well as the probabilistic approaches that have been adapted to identify dominant directions in more cluttered scenes [4].

Newer line detectors like LSD [5] have increased the precision and the recall for detecting edges. Additionally, consensus-based methods such as MLESAC [6] and PROSAC [7], in conjunction with scene priors like Manhattan [8] and Atlanta worlds [9], have improved the reliability of VP estimation. Still, purely geometric methods can struggle with high noise at intersections and with sparse sampling. In this regard, noise resilient methods such as learning-based approaches [10,11] and differentiable models [12,13] are likely to perform better. However, this typically comes at a high cost in terms of the amount of data required (which can cause a lack of generalization) for the system to perform well on previously unseen data.

**Figure 1:**
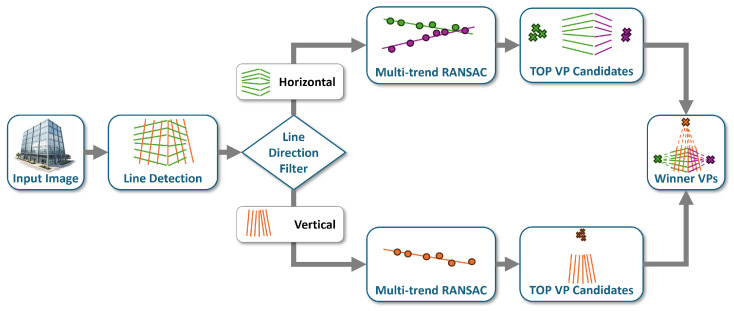
Block diagram of the proposed DRR-MMT-VPD pipeline.

These issues contributed to the development of the Dual RANSAC with Rescue Midpoint-based Multi-Trend Vanishing Point Detection (DRR-MMT-VPD) framework. As shown in Figure 1, our approach avoids hard constraints such as enforcing orthogonality between vanishing directions, which is typical in strictly Manhattan-world formulations. Instead, the method is built on a simple geometric cue: the strong linear relationship between the orientation of a line segment and its position in the image. The proposed method benefits from this relationship by applying a midpoint-based RANSAC stage [14] directly to the segment attributes (midpoints and orientations) before computing pairwise intersections. This early consensus step helps isolate dominant directional trends while filtering out many spurious segments that would otherwise degrade intersection clustering.

The main contributions of this research can be summarized by the following:

- A midpoint-based multi-trend RANSAC formulation that operates on line segment midpoints and orientations, allowing the separation of directionally dominant groups prior to the generation of candidate vanishing points.
- A candidate construction and evaluation stage where several operations are performed, including pairwise intersection analysis, adaptive DBSCAN clustering [15], confidence, and length-based ranking [16].
- A robust final selection strategy in which MSAC-based evaluation [6] is used to geometrically evaluate consistency and select the most reliable hypothesis, while a rescue mechanism re-inspects failed or weak trends to retrieve potential vanishing point candidates when the primary selection stage is insufficient.

Our framework is evaluated against two standard benchmarks: the York Urban Dataset (YUD) [17] and the Toulouse Vanishing Points Dataset (TVPD) [18], using standard angular-error metrics to demonstrate its accuracy and reliability. Experimentally, the proposed framework shows the strongest performance under strict angular-error thresholds on both benchmarks. For angular accuracy @1° a recall of 82% is achieved on the YUD and 83% on the TVPD.

The remainder of this paper is organized as follows. Section 2 reviews related work on VP detection methods, including consensus-based and learning-based approaches. Section 3 describes the proposed DRR-MMT-VPD pipeline in detail. Section 4 presents the experimental setup and datasets. Section 5 provides the results of the evaluation and comparisons of the proposed method against different benchmarks. Section 6 concludes this paper and outlines the directions for future research.

## 2. Related Work

Research on vanishing point (VP) detection has spanned several different yet interrelated paths, including classical geometric consensus, clustering methods and, most recently, methods derived from deep learning. This section features the key developments in the areas of line detection and extraction, multi-model clustering, robust model fitting, and learning-based VP detection, and positions our method in this landscape.

### 2.1. Line Detection

The reliability of the estimation model is determined by the line segments from which the model is constructed. Among line segment detectors, the LSD [5] is a good candidate due to its efficiency and its statistical approach to the problem of controlling false positives. However, line extraction is prone to failure in low-contrast, blurry, and noisy environments. When environments become fragmented or even disappear, they may impede reasoning and consensus about intersections. In this regard, in our framework, a local contrast enhancement and bilateral smoothing are implemented to produce longer and better localized segments to improve consensus in the downstream tasks. Several supportive techniques use Hough-type voting to extract line features, where edge points are mapped to a (ρ,θ) parameter space. Probabilistic and progressive variants [2,3,4] can affect the extent of computation and the suppression of spurious peaks while preserving strong baselines for man-made scenes. Additionally, lightweight detectors such as EDLines [19] provide rapid line extraction through direct chaining of edge information. In all these approaches, the most important point holds: more robust and less fragmented line evidence leads to more robust VP hypotheses.

### 2.2. Robust Consensus Estimation

Images with real-world data have noise, occlusions, missing line segments, and erroneous detections. Thus, VP estimation requires robust fitting. RANSAC solves this issue by iterative sampling of minimal sets, creating a fitting hypothesis, and determining the model with maximal inlier support with respect to a threshold of residuals [14]. Many adaptations of RANSAC focus on improving the efficiency or the scoring of this model selection problem. For instance, MSAC and MLESAC focus on inlier counting. MSAC applies a truncated loss, and MLESAC uses a mixture of likelihood of inliers and outliers to score hypotheses [6]. PROSAC also improves the efficiency of this process by sampling high-quality hypotheses that have been pre-ordered [7].

### 2.3. Multi-Model Clustering

Most scenes possess multiple dominant directions, so it is important that VP detection splits candidates into consistent sets. J-Linkage [20] and its continuous relaxation T-Linkage [16], are examples of preference-set methods that cluster points supporting the same hypothesis, and both can retrieve multiple models without requiring prior knowledge of the number of VPs. Their principal drawback is cost: J-Linkage can be slow, and its performance can drop for noisy or nearly collinear configurations. As a more efficient alternative, density-based clustering (DBSCAN) [15] groups intersections by spatial density while rejecting outliers. The application of DBSCAN clustering in VP-specific environments has been experimented with earlier, such as in omnidirectional images [21]. In our pipeline, however, DBSCAN is applied to compact clusters prior to the consensus and selection phase in order to cluster VPs from intersection analysis and oriented trend regression.

### 2.4. Vanishing Point Estimation Methods

Initial geometric techniques approximated VPs by either direct line crossings or by using the projective topology of the architectural scene [22]. To regularize the problem in man-made environments, researchers often invoke Manhattan-world or Atlanta-world assumptions. The Manhattan assumption is frequently evaluated on urban datasets [17], while the Atlanta-world model generalizes to multiple horizontal directions via Expectation-Maximization (EM) frameworks [9]. The applied priors often improve the model’s stability, but they reduce the model’s adaptability if the scene does not conform to the assumed structural details. In contrast, our method does not involve this trade-off. It remains agnostic to any assumption, using midpoint parameterization, dual consensus, and density-based clustering, and does not impose any architectural assumptions.

### 2.5. Learning-Based Approaches

Learning-based approaches to vanishing point (VP) detection generally follow two main directions. Some methods predict vanishing points directly from image content, whereas others learn how to generate, sample, or rank hypotheses more effectively. DeepVP [11], for example, is an end-to-end method designed for VP detection in street-view imagery. By contrast, CONSAC [23] does not predict vanishing points directly, but learns a context-dependent sampling distribution for robust multi-model fitting. In this sense, CONSAC should be regarded as a network-guided hypothesis-generation method rather than a purely geometric baseline. Such learning-based strategies can improve structured sampling and robustness in complex scenes, but they typically rely on supervised training and may generalize less reliably beyond the domains seen during training. Most recent VP methods employing machine learning have focused on geometry-detected feature learning. NeurVPS [24] proposes conic convolution to combine image evidence along line families that meet at the same vanishing points. In a similar approach, explicit geometric priors are incorporated into the neural network. In particular, Lin et al. [25] introduce the concept of a trainable Hough transform and Gaussian sphere mappings, allowing the projective structure to regularize the deep features and enhancing the degree of robustness against the variations in the dataset and the non-Manhattan-type scenes. These techniques are of particular importance as they represent a more advanced shift from the general image-based approaches to more integrated geometrical reasoning into the structures. This tendency has been further extended in models based on Transformers. VPDETR [26] considers VP detection as a set-prediction concern, minimizing the necessity of manually crafted post-processing, and generalizing the method to both Manhattan and non-Manhattan scenes. VPTR [27] proposes a real-time, end-to-end Transformer architecture for VP detection and prioritizes direct prediction of vanishing points. In contrast to previous approaches based on convolution neural networks (CNNs), methods based on Transformers experience improved modeling for global context. On the other hand, they bring increased complexity for the model along with the conventional dependence on a well-defined set of labeled data and training. Recent approaches show how contemporary VP research encompasses both end-to-end learned prediction and geometry-aware learning approaches. DeepVP fetches vanishing points from image content, CONSAC learns to sample hypotheses more effectively, and hybrid methods, like Hough space and spherical accumulators, use geometric structures learned during training. In contrast to these approaches, DRR-MMT-VPD integrates precise line extraction [5], consensus-based fitting [14], and density-based clustering [15] in a single, unified, training-free framework.

This geometric and rule-based design’s main contribution is a multi-trend formulation based on midpoints, which separates multiple dominant directional groups prior to the generation of candidate vanishing points. This early separation results in well-conditioned hypotheses, which aid in subsequent clustering, ranking, and consensus, while maintaining traceability and interpretability, and avoiding supervised learning and restrictive scene priors.

Table 1 summarizes all methodological families reviewed in this section, showing the main limitations and the corresponding benefit offered by our framework.

Unlike these methods, DRR-MMT-VPD combines accurate line extraction [5], consensus-based fitting [14], and density-based clustering [15] in a unified training-free pipeline. This geometric and rule-based design preserves traceability and interpretability, while avoiding supervised learning and restrictive scene priors. Table 1 summarizes all methodological families reviewed in this section, showing the main limitations and the corresponding benefit offered by our framework.

In contrast to these approaches, DRR-MMT-VPD integrates precise line extraction [5], consensus-based fitting [14], and density-based clustering [15] in a single, unified, training-free framework. This method is geometric and rule-based; it avoids supervised learning and confining scene assumptions, and preserves traceability and interpretability.

All of the methodological families that have been reviewed in this section, including our proposed framework, are summarized in Table 1, along with their major shortcomings and the associated advantages of DRR-MMT-VPD for each.

## 3. Proposed Method

In this section, Dual RANSAC with Rescue Midpoint-based Multi-Trend Vanishing Point Detection (DRR-MMT-VPD) is introduced as a flexible and accurate pipeline for vanishing point estimation in complex images. It combines adaptive statistical analysis and geometric estimation with a meticulously ordered sequence of processes, each of which refines the distribution input and enforces geometric consistency with the input for the next stage.

The sequence of steps, which range from local edge detection to global consensus, facilitates information flow and vanishing point detection and guarantees correspondence to the dominant structural trends in the scene. Figure 1 shows the overall architecture of the proposed framework, starting from the input scene and ending with the selection of the vanishing points. The overall procedure of the proposed pipeline is summarized in Algorithm 1, while the main components of the pipeline are described in detail in the subsequent paragraphs.
**Algorithm 1:** DRR-MMT-VPD **Input**: Image *I* **Output**: Vanishing points *V* L← Detect and preprocess lines (Section 3.1); Split *L* into $\left\{L_{H},L_{V}\right\}$;
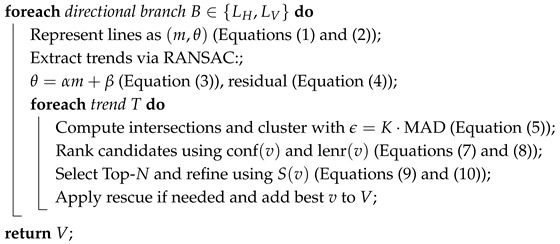


### 3.1. Line Detection and Preprocessing

**Figure 2:**
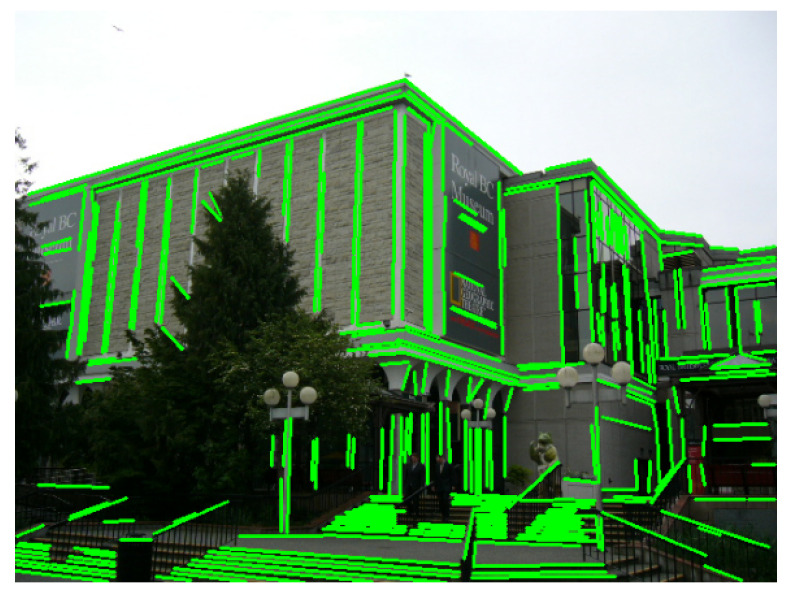
LSD-based line detection: detected segments (green) overlaid on the input scene.

In the first stage of the DRR-MMT-VPD pipeline, line segments are extracted (Figure 2) using the line segment detector (LSD) [5], a sub-pixel line segment detector that uses a probabilistic model, with respect to Otsu, which minimizes false positives and is capable of identifying line segments in perspective-dominated scenes. Prior to line detection, certain preprocessing techniques are employed, including contrast enhancement and adaptive binarization, to improve edge localization and detection when the input image is of low contrast or is unevenly lit. A summary of the preprocessing parameter is documented in Table 2. Specifically, Contrast-Limited Adaptive Histogram Equalization (CLAHE) [28] can be used to boost local contrast, in addition to adaptive thresholding [29] to sharpen edges when illumination is uneven. Post-processing can be optional, and the grayscale image undergoes the application of two filters.

Preserving edge discontinuities, the bilateral filter [30] is intended for smoothing local intensity variations, whereas the Gaussian filter [31] is employed for the suppression of remaining high-frequency noise. This results in a clean and uniform image, retaining the significant structural details. To minimize unstable detections and to retain only the geometrically relevant attributes, segments shorter than a pre-set minimum length are discarded.

At this point, after the line segments are extracted, directional filtering is performed, and the segments are sorted by direction. Only segments that fall within the established vertical and horizontal angle cutoffs are retained for processing in successive steps of the pipeline. The primary parameters used in the preprocessing and filtering steps are provided in Table 2.

**Figure 3:**
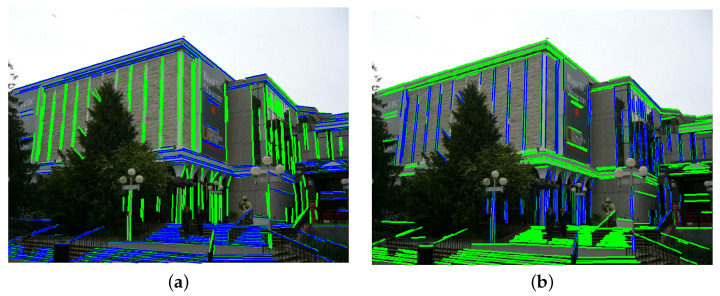
Directional line filtering results used by our pipeline applied on detected line segments (green). (**a**) Horizontal-filtered lines (blue). (**b**) Vertical-filtered lines (blue).

To keep the lines organized and correctly oriented, the lines are first sorted in order of their endpoints, then the following filters are applied:

- Horizontal filtering: Retains lines around 0° or 180° and within a horizontal angular threshold (Figure 3a);
- Vertical filtering: Retains lines around 90° and within a vertical threshold (Figure 3b).

Segments located outside of these ranges are eliminated.

This split-in-direction gives two independent sets of input lines, one horizontal and one vertical, which are then routed separately through the DRR-MMT-VPD pipeline. Running the pipeline on each set decreases orientation ambiguity and increases the speed of convergence in subsequent consensus stages such as RANSAC [14].

After directional filtering, DRR-MMT-VPD switches from segment endpoints analysis to a midpoint–orientation representation, which enhances statistical stability for geometric estimation. A segment’s midpoint provides a location reference that is less affected by factors such as occlusion, segmentation, truncation errors, gradient shifts, illumination changes or endpoint localization errors [1], while the segment endpoints are much more affected by the aforementioned characteristics as opposed to endpoint localization errors [4]. This method of midpoint-based parameterization develops a more consistent and uniform distribution of the samples in space, which positively affects the conditioning of regression, as well as the reliability of consensus [32]. Consider each detected line segment, defined by endpoints $\left(x_{1},y_{1}\right)$ and $\left(x_{2},y_{2}\right)$, is represented by its midpoint *m* and orientation θ:

(1) $$m=\left(\frac{x_{1}+x_{2}}{2},\frac{y_{1}+y_{2}}{2}\right),$$

(2) $$\theta =\arctan\left(\frac{y_{2}-y_{1}}{x_{2}-x_{1}}\right).$$

This formulation diminishes the redundancy among symmetric line pairs and helps to simplify the computation of the orientation histogram and trend clustering. In both the horizontal and vertical cases, when angles are mapped to either $\left[0^{{}^{\circ}},180^{{}^{\circ}}\right]$ or $\left[-90^{{}^{\circ}},90^{{}^{\circ}}\right]$, lines with opposite directions but identical orientations are treated equivalently. Without this normalization step, consensus methods like RANSAC [14] can incorrectly split them into different models [8]. However, with normalization, both polarities are combined, which enhances the inlier aggregation and the vanishing point detection from rays that cross over from the opposite sides of the image plane.

### 3.2. RANSAC over Midpoint–Orientation Space for Trend Estimation

From the previous step, the horizontal and vertical line sets are estimated separately, and midpoint coordinates, in addition to the line orientation angle, are jointly computed for each line. Lines in each directional set are represented by $\left(m_{j},\theta_{j}\right)$ pairs in the midpoint–orientation space that derive joint spatial and angular information.

Our parametric approach models the orientation variation as a linear regression in the midpoint–orientation space inspired by the perspective rectification framework in [33]. Unlike approaches relying on histogram voting or angular clustering [8], our model retains the spatial correlation of the position and direction of the line segment, which is a crucial feature for perspective images. By applying RANSAC [14] independently to each directional line set, the dominant orientation inlier subsets can be extracted, thus describing the structural arrangement of the lines, which is the foundation for calculating the vanishing point in the following steps.

Based on the midpoint–orientation pairs $\left(m_{j},\theta_{j}\right)$ corresponding to each directional line set, dominant orientation trends are extracted as 1-D regressions in the (m,θ) plane [33]:

(3) θ=αm+β,

where θ is the line orientation (in degrees), and *m* is the centroid coordinate used for regression (in pixels), defined as m=x for horizontal configurations and m=y for vertical ones. α is the slope (degrees per pixel) that describes how the orientation varies with *m*, and β is the intercept (degrees) representing the orientation when m=0. Angles are normalized to the selected range (e.g., $\left[-90^{{}^{\circ}},90^{{}^{\circ}}\right]$ or $\left[0^{{}^{\circ}},180^{{}^{\circ}})\right]$ before fitting. For the evaluation of the RANSAC model, inliers are identified by the angular residual criterion.

(4) $$\left|\;\theta_{j}-\left(\alpha \;m_{j}+\beta \right)\;\right|\;<\;\tau_{\mathrm{tr}}\;,$$

where $\tau_{\mathrm{tr}}$ is an angular threshold (in degrees). Angle normalization (e.g., wrapping to $\left[-90^{{}^{\circ}},90^{{}^{\circ}}\right]$ follows the chosen orientation scale, with $\tau_{\mathrm{tr}}$ as an angular threshold (in degrees). The normalization of angles (e.g., wrapping to $\left[-90^{{}^{\circ}},90^{{}^{\circ}}\right]$ follows the chosen orientation scale. The angular residual presented in (4) measures how well the direction of a segment matches the trend, rather than how close the points are in the image.

**Figure 4:**
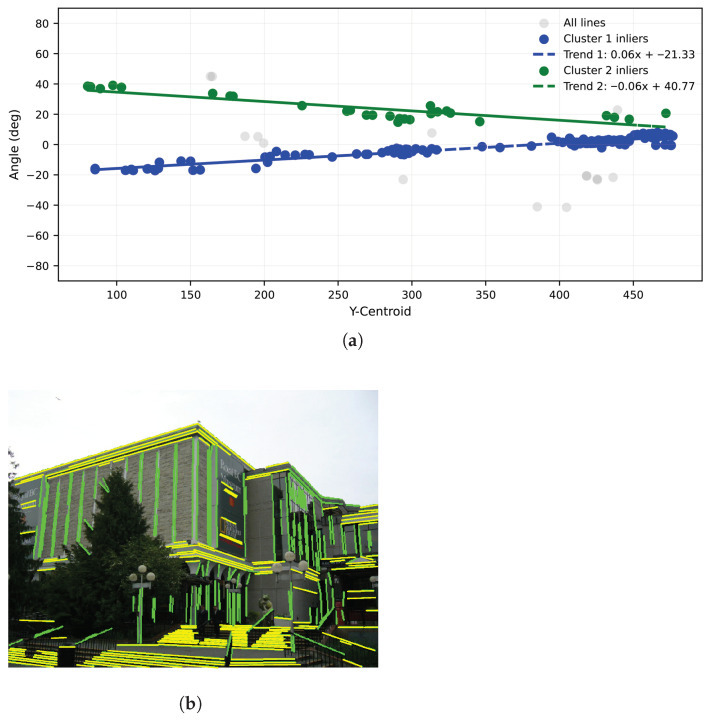
Trend fitting and inlier lines for the horizontal case. (**a**) Horizontal two-trend RANSAC fit. (**b**) Horizontal inlier lines (yellow).

**Figure 5:**
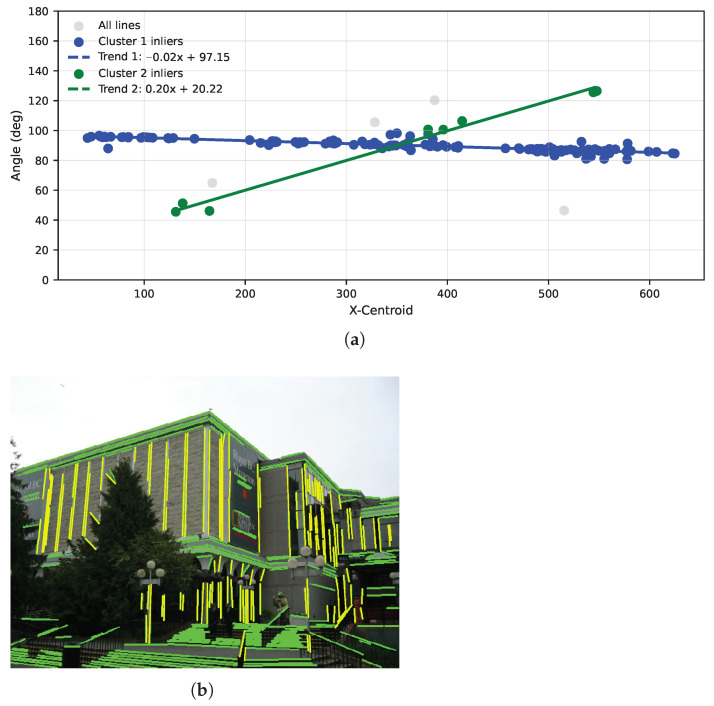
Trend fitting and inlier lines for the vertical case. (**a**) Vertical two-trend RANSAC fit. (**b**) Vertical inlier lines (yellow).

To capture multiple orientation modes within each case (horizontal or vertical), the fitting procedure is run iteratively: the dominant trend is estimated, its inliers are removed, and the process repeats on the residuals. Up to two trends per either horizontal or vertical direction are typically sufficient to represent the main directional structure without imposing Manhattan constraints. The iteration ends when the remaining data are insufficient for a stable fit. Figure 4 and Figure 5 illustrate the two extracted trends and their respective inlier line groups for the horizontal and vertical cases, respectively, demonstrating the ability of the proposed method to meaningfully distinguish different dominant directional modes and to supply well-defined subsets for later stages of vanishing point estimation.

For each discovered trend *t*, the following are retained: (i) the corresponding parameters $\left(\alpha_{t},\beta_{t}\right)$ from (3), (ii) the index set of inlier segments $I_{t}=\{j$: (4) holds}, and (iii) the associated subset of line segments. These inlier subsets form the inputs for the computation of pairwise intersection and for the generation of the clustered Top-*N* vanishing point candidates in the next stage.

### 3.3. Intersection Computation and Clustering

Once the dominant orientation trends are determined for each horizontal and vertical direction, the inlier line segments from each trend are then employed to derive potential vanishing point (VP) candidates. Each segment, defined by its endpoints, is converted to a homogeneous line representation, and all non-parallel pairs are intersected to obtain image-plane intersection points [1], which serve as geometric hypotheses of where 3D parallel directions meet.

Due to localization errors, occlusions, and detection noise, intersections rarely coincide exactly but form compact spatial clusters around the true VP. Instead of averaging, which is sensitive to outliers, a density-based clustering method is applied to isolate consistent intersection subsets. DBSCAN (Density-Based Spatial Clustering of Applications with Noise) [15] is used since it groups dense regions without pre-specifying the number of clusters and naturally rejects sparse noise, properties well suited to non-Gaussian intersection clouds [21].

The neighborhood radius ε is adaptively determined from the spread of the intersection coordinates using the median absolute deviation (MAD) [34]:

(5) $$\varepsilon =K\cdot \mathrm{MAD}(\left\{x_{i},y_{i}\right\}),$$

where *K* is a scaling constant that controls the compactness of the cluster. The MAD provides a robust scale dispersion measure, allowing ε to self-adjust to image resolution and scene geometry and act as the effective merging radius for candidate VPs.

Each resulting cluster represents a potential region of a vanishing point. Its median location is taken as the initial VP estimate and passed to the subsequent refinement and ranking stage, yielding a compact, noise-filtered set of geometrically consistent VP hypotheses.

### 3.4. Candidate Ranking and Top-N Selection

After grouping intersection points into areas associated with a vanishing point (VP), the next task is to evaluate the potential of each candidate to explain the perceived line structure and keep the most representative hypotheses for further processing. Each VP candidate is assessed based on two scoring criteria: confidence, which is the number of line segments in agreement with the VP candidate direction, and length ratio, which is the total length of line segments advocating that candidate divided by the total length of all line segments in the corresponding trend. Both metrics depend on the directional angles created between each candidate VP and the associated supporting line set, which describes the directional agreement and the geometric support for each candidate VP. This approach embodies the consensus principle of RANSAC [14] and its M-Estimator variants, notably MSAC and MLESAC [6], which quantify model complexity based on data agreement, not solely on a single model’s residuals. Using these two metrics as ranking criteria, the proposed method identifies the candidates that best represent the dominant directional organization of each trend.

Let $L=\{\ell_{1},\ldots ,\ell_{m}\}$ be the set of line segments for the current trend, τ>0 be the angular inlier threshold (degrees), and let ∠(ℓ,v) denote the acute angle between the orientation vector of each segment and the line from its midpoint to the candidate vanishing point (VP) *v*. The inlier set for *v* is defined as [35,36]

(6) $$I\left(v\right)\;=\;\left\{\ell \in L:\;∠(\ell ,v)<\tau \right\}.$$

where each candidate *v* is evaluated using two normalized metrics:

Confidence score (inlier fraction): The fraction of line segments in the current trend that are consistent with the candidate VP, in the RANSAC consensus sense [14].

(7) $$\mathrm{conf}\left(v\right)\;=\;\frac{\left|I(v)\right|}{\left|L\right|}.$$

Length ratio (support strength): Letting ∥ℓ∥ denote the Euclidean length of segment *ℓ*, this score measures the fraction of the total segment length in the current trend that is contributed by the line segments supporting candidate *v*, as a length-weighted support measure motivated by earlier VP formulations [35,37].

(8) $$\mathrm{lenr}\left(v\right)\;=\;\frac{\sum_{\ell \in I(v)}∥\ell ∥}{\sum_{\ell \in L}∥\ell ∥}.$$

Both conf(v) and lenr(v) lie in [0,1], where the first measures the proportion of consistent segments and the second their cumulative length contribution.

**Figure 6:**
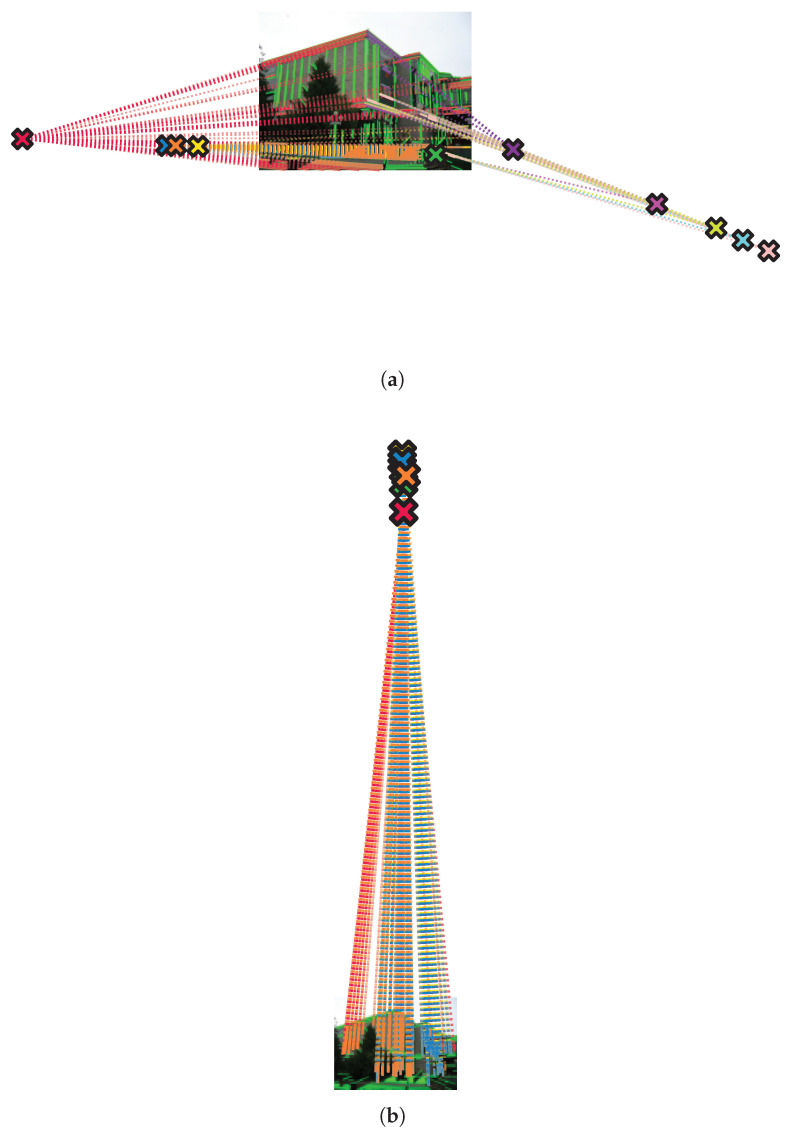
Horizontal and vertical Top-N VP candidate for each dominant trend shown as x marks. (**a**) The Top-N VPs for dominant horizontal trends. (**b**) The Top-N VPs for dominant vertical trends.

Candidates are lexicographically ranked by $\left(\mathrm{conf}(v),\;\mathrm{lenr}(v)\right)$, and the Top-*N* candidate VPs per trend are passed to the subsequent refinement stage (Figure 6).

At this point, the method does not commit to a single vanishing point; rather, it preserves a compact pool of the most geometrically plausible hypotheses, together with their supporting line sets, so that MSAC-based verification and rescue arbitration can operate on a reduced and better conditioned candidate space.

### 3.5. RANSAC-for-a-Point with MSAC Scoring

After ranking and retaining the Top-N vanishing point (VP) hypotheses per trend, this stage selects the most representative VP. A second consensus-based refinement operates directly on the candidate pool to address redundancy, overlap, and local inconsistencies from intersection clustering. This refinement operates in the VP hypothesis space, trying to detect the candidate that maximizes directional consensus with the supporting lines segment. The scoring follows an MSAC-style formulation [6], where angular deviations are quadratically penalized up to a threshold τ (degrees). Each line $l_{j}$ contributes proportionally to its length-based weight $w_{j}={∥l_{j}∥}^{\gamma }$, with γ>0 adjusting the influence of longer segments. A secondary RANSAC-based consensus stage is performed to identify the final vanishing point (VP) by refining and evaluating the most plausible hypotheses of Top-N candidates for each trend. The procedure is as follows:

1. Sampling: Preferentially sample higher-quality candidates while maintaining diversity (prefix-style sampling over the Top-N pool), following the progressive ranked-sampling idea of PROSAC [7].
2. Hypothesis refinement: For a seed candidate *v*, collect its local neighborhood (optionally by pixel or angular proximity), merge its supporting lines, and refine *v* using least-squares fitting.
3. Inlier evaluation: A line $l_{j}$ is an inlier for *v* if its angular deviation $\theta_{j,v}$ from the vector connecting its midpoint to *v* is below τ (degrees).
4. MSAC-style scoring: Each hypothesis *v* is scored using a length-weighted objective inspired by the MSAC/MLESAC robust consensus framework [6]:

(9) $$S_{\mathrm{MSAC}}\left(v\right)\;=\;\frac{1}{\sum_{j}w_{j}}\sum_{j}\max\;\left(0\;,\;1-{\left(\frac{\theta_{j,v}}{\tau }\right)}^{2}\right)\;w_{j}\;,$$

where $w_{j}={∥l_{j}∥}^{\gamma }$ is the length-based weight. To stabilize selection, the final score adds an inlier fraction bonus and a dispersion penalty:

(10) $$S\left(v\right)\;=\;S_{\mathrm{MSAC}}\left(v\right)\;+\;\beta \cdot \frac{\left|I(v)\right|}{\left|L\right|}\;-\;\lambda \cdot \frac{\mathrm{std}\;\left({\left\{\theta_{j,v}\right\}}_{j\in I(v)}\right)}{\tau }\;,$$

where I(v) is the inlier set and *L* is the total line set; β>0 controls the inlier fraction bonus, and λ>0 represents the angular dispersion penalty. The highest-scoring hypothesis is selected as the final winner VP of the trend. This formulation keeps the statistical essence of RANSAC [14] and, at the same time, adds a continuous data-weighted scoring mechanism built on an MSAC/MLESAC-style basis [6].

**Figure 7:**
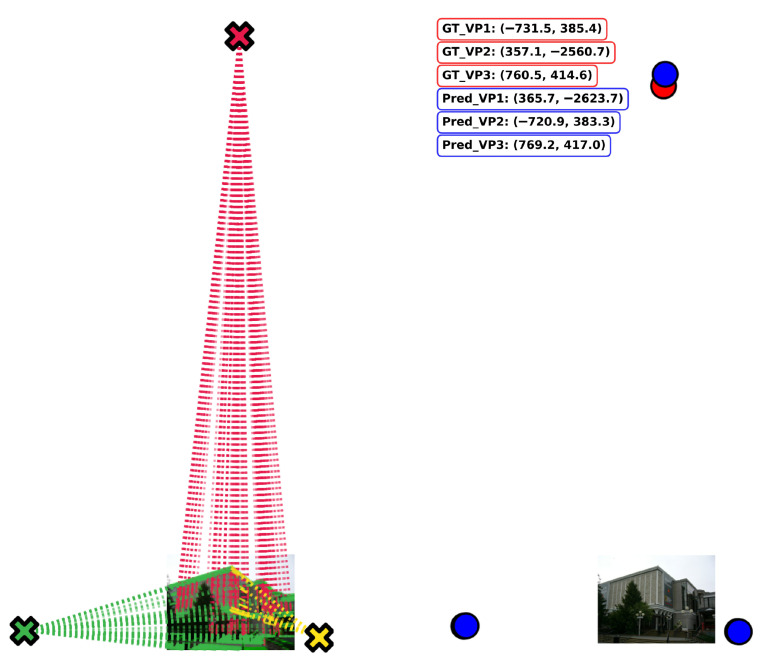
Visualization of the winner vanishing points (blue) and their correspondence with the ground-truth vanishing points (red). The matched pairs are GT_VP1 ↔ Pred_VP2, GT_VP2 ↔ Pred_VP1, and GT_VP3 ↔ Pred_VP3.

This stage outputs one well-localized VP per directional trend, consolidating multiple hypotheses into a single geometrically consistent solution. Figure 7 shows how all winner VPs plotted on the original image (left) with their corresponding coordinates compared with the groundtruth VPs (right).

### 3.6. Rescue Arbitration

In some cases, a trend may not succeed in identifying a valid vanishing point (VP) during the standard RANSAC–MSAC stage due to many restrictions, such as an insufficient number of inliers, high angular dispersion, or restrictive thresholds. Instead of discarding that trend, the pipeline triggers a rescue arbitration step that reuses the same RANSAC-for-a-point framework under relaxed constraints. This process runs independently per trend; if a trend corresponding to one of the two directional groups (either horizontal or vertical) fails to converge, then only the candidate pool of that specific trend is re-evaluated, preserving the consistency of the other trend in the same directional group and preventing error propagation.

The rescue stage uses the same initial sampling and scoring logic, but applies softer angular thresholds, and requirements for inliers are less stringent. In this stage, scoring and refinement are done according to the MSAC (M-Estimator Sample Consensus) formulation [6] to keep methodical consistency. The candidates are re-scored and rank-ordered based on their weighted MSAC scores.

The original result for all dominating trends is preserved unless, in the case of the rescued trend, the score of the strongest rescued candidate surpasses that candidate score from the main stage, in which case, the main stage score is updated to the rescue stage score.

This type of selective replacement ensures that each trend is assigned, at most, one unambiguous VP, thus preserving the coherence of the previous consensus variant.

To summarize, the DRR-MMT-VPD pipeline, in principle, translates the image features into validated VPs through a sequence of detection, filtering, midpoint-based regression, and consensus-based selection. Each stage refines the statistics from the previous stage, reinforcing all the geometric evidence and ensuring ultimately that each trend results in a single valid VP for the quantitative evaluation.

The algorithm uses an initial set of tunable parameters, categorized by stage function, starting from detection, trend estimation, and consensus clustering, arriving at the rescue arbitration stage. The configuration protocol and validation strategy are found in Section 4.2.

## 4. Experimental Setup

In this section, the experimental protocol adopted to evaluate the proposed algorithm is outlined. The presentation begins with the datasets and evaluation metrics used in the experiments, followed by a summary of the baseline methods considered for comparison with DRR-MMT-VPD.

This section details the experimental procedure used to assess the proposed algorithm. It begins by introducing and defining the datasets and metrics used to evaluate the algorithm, and ends with a brief description of the baseline methodologies used in the DRR-MMT-VPD evaluation process.

### 4.1. Datasets

For a comprehensive evaluation, our method is tested over two public datasets widely used for vanishing point detection: the York Urban Dataset (YUD) [17] and the Toulouse Vanishing Points Dataset (TVPD) [18]. They span across a broad range of scene types, offering not only a cluttered urban outdoor scene type but also an organized indoor environment scene type, thereby providing a more balanced assessment of the proposed technique across different visual scenarios and scene geometries.

YUD: The YUD is a widely used benchmark for vanishing point detection. It consists of outdoor urban images with prominent man-made structures, where parallel lines often show clear perspective convergence. The dataset provides ground-truth VPs for three dominant, mutually orthogonal directions, largely reflecting a Manhattan-world scene model.

TVPD: This dataset, commonly referred to as Toulouse, includes pictures taken in Manhattan-like scenes using an iPad Air 1. A characteristic of TVPD is that it includes Inertial Measurement Unit data synchronized with the camera for reliable estimation of ground-truth vanishing points. The dataset emphasizes structured spatial layouts, which makes it suitable for evaluating methods that detect mutually orthogonal vanishing points. The main characteristics of the YUD and TVPD are summarized in Table 3.

### 4.2. Parameter Configuration and Validation

All adjustable parameters of the DRR-MMT-VPD pipeline can be organized into four groups, corresponding to their operational role within the proposed pipeline:

Detection Parameters: This set of parameters controls the fine-tuning and stability of the early vision front-end, which includes the parameters of contrast enhancement, edge thresholding, and minimum line-length control by the line detector. These parameters control the balance between noise control and the sufficiency of the geometric data extracted from the image.

Trend Estimation Parameters: These parameters control the geometric consensus mechanism that is performed by RANSAC, specifically the inlier angle tolerance, the threshold for the distance of the residual, and the maximum number of iterations. They define the reliability of dominant trend extraction and ensure that only structurally coherent line clusters contribute to candidate vanishing points.

Consensus and Clustering Parameters: This set of parameters determines how intersection points are aggregated using the DBSCAN algorithm and spatial gating rules, with parameters such as the neighborhood radius and minimum cluster size controlling the granularity of intersection grouping. These settings manage how well the method differentiates between various vanishing directions while preventing unuseful splitting into additional groups.

Rescue Arbitration Parameters: The rescue stage is a secondary fallback that only triggers when the first pass fails to select a credible winner vanishing point. Rather than repeating the same decision-making process, it expands the search and relaxes the acceptance criteria, so that valid hypotheses are not rejected due to challenging cases such as partial occlusion, uneven line support, or heavy clutter. During this pass, candidates are reclassified, then re-scored using less restrictive angular and support criteria over a larger candidate set. When enabled, a geometric light-weight prior positively influences solutions and enforces separation between directions. The result from the rescue stage will only replace the previous result if it finds a candidate that has more support. Otherwise, the original result will be retained.

### 4.3. Implementation Details

The entire DRR-MMT-VPD framework has been implemented using Python 3.13.1, mainly using OpenCV 4.11.0, NumPy 2.2.3, and scikit-learn libraries. All experiments were conducted on a laptop with an AMD Ryzen 7 5700U and 16 GB RAM. RANSAC-based trend estimation and the MSAC (M-Estimator Sample Consensus) variant were implemented as previously described in Section 3, and DBSCAN was used for intersection clustering with adaptive scaling as explained in Section 4.2.

To ensure similar behavior in all datasets, all random seeds were the same, and the same software configuration was used on both datasets (YUD and TVPD). The modularity of the proposed pipeline allows independent execution and evaluation of all its components, thereby facilitating performance tracking and benchmarking.

### 4.4. Evaluation Metrics

In the purpose of evaluating DRR-MMT-VPD, a set of metrics was adopted to extract three practical aspects of vanishing point detection: localization accuracy, detection coverage, and scene-level reliability. Let the ground-truth vanishing point be $\mathrm{gt}\_\mathrm{vp}=(x_{g},y_{g})$ and the predicted vanishing point be $\mathrm{pred}\_\mathrm{vp}=(x_{p},y_{p})$; both are expressed in pixel coordinates. For an image of width *W* and height *H*, the image center is

(11) $$c=\left(\frac{W-1}{2},\frac{H-1}{2}\right).$$

where center-referenced vectors are formed:

(12) $$v_{gt}=\mathrm{gt}\_\mathrm{vp}-c\;\mathrm{and}\;v_{p}=\mathrm{pred}\_\mathrm{vp}-c.$$

#### 4.4.1. Median Angular Error (Median AE)

A standard metric for evaluating vanishing point detection is the angular error (AE). AE measures, in degrees, the angle between a predicted unit direction vector $v_{p}$ and its matched ground truth $v_{gt}$. Following the prior vanishing point literature, the angular error between $v_{gt}$ and $v_{p}$ is computed as in Equation (13) and reported in degrees [38]:

(13) $$AE\;\left(v_{p},v_{gt}\right)=\frac{180}{\pi }\;\arccos\left(\;\left|\;v_{p}\cdot v_{gt}\;\right|\;\right).$$

The Median AE represents the median error across all successfully matched pairs. A lower Median AE indicates higher accuracy.

#### 4.4.2. Precision and Recall

These metrics evaluate the quality of the point-level detection. Precision measures the reliability of the predictions, i.e., the ratio of true positives to total predictions, whereas recall measures the completeness, i.e., the ratio of recovered ground-truth VPs to the total number of ground-truth VPs, following the standard definitions of precision and recall [39]. Both are evaluated at a threshold θ (e.g., $3^{{}^{\circ}}$), using the Hungarian algorithm [40] on the AE cost matrix

(14) $$C_{ij}=\mathrm{AE}\;\left(\;\mathrm{gt}\_{\mathrm{vp}}_{i}\;,\;\mathrm{pred}\_{\mathrm{vp}}_{j}\;\right).$$

This ensures a one-to-one assignment between ground-truth and predicted VPs for each image.

The resulting matched pair errors $E=\{e_{k}\}$ are then aggregated over the evaluation set to report (i) the Median AE and (ii) the number of matched pairs. Precision and recall at a threshold τ are defined as follows [39]:

(15) $$\mathrm{Recall}\left(\tau \right)=\frac{\left|\{e_{k}<\tau \}\right|}{N_{\mathrm{GT}}},\;\mathrm{Precision}\left(\tau \right)=\frac{\left|\{e_{k}<\tau \}\right|}{N_{\mathrm{Pred}}}.$$

Here, $N_{\mathrm{GT}}$ and $N_{\mathrm{Pred}}$ denote the total numbers of ground-truth and predicted VPs in the evaluation set.

#### 4.4.3. Image Success Rate

The image-level success rate is also reported at threshold τ, a standard evaluation measure in vanishing point detection under angular-error tolerance [41]. In our formulation, this metric is defined in a strict image-level sense as the fraction of images for which all ground-truth VPs are matched, and the maximum matched AE does not exceed τ, as given in Equation (16). This is particularly relevant for Manhattan-world applications, where successful recovery requires the complete VP configuration of the scene [41]:

(16) $$\mathrm{Success}\left(\tau \right)=\frac{1}{N_{\mathrm{img}}}\sum_{i=1}^{N_{\mathrm{img}}}1\left[\;\left|E_{i}\right|=N_{\mathrm{GT},i}\;,\;\max\left(E_{i}\right)\leq \tau \;\right].$$

#### 4.4.4. Area Under the Curve (AUC)

The Area Under the Curve (AUC) is the normalized integral of a performance curve, summarizing how a metric evolves as the allowed angular-error threshold increases. In our evaluation, the recall AUC, the image success rate AUC, and the precision AUC are computed by integrating the corresponding curves on the thresholds (from 0 to $10^{{}^{\circ}}$), where the y-axis gives the proportion of correct detections at each threshold. Therefore, higher AUC values indicate better overall performance and better robustness across the full range of error tolerances. As detailed in Equation (17), for a cap *T* (in degrees), ${\mathrm{AUC}}_{0:T}$ is computed via trapezoidal integration of uniformly sampled thresholds:

(17) $${\mathrm{AUC}}_{0:T}=\frac{1}{T}\int_{0}^{T}\mathrm{Recall}\left(t\right)\;dt.$$

#### 4.4.5. Angle Accuracy (AA)

Angle Accuracy (AA) is the proportion of detections that satisfy an angular-error tolerance *t* (e.g., $1^{{}^{\circ}}$, $2^{{}^{\circ}}$, $5^{{}^{\circ}}$, $10^{{}^{\circ}}$). AA is independently reported at each threshold (AA@*t*) and therefore does not aggregate performance between tolerances; instead, it provides a direct snapshot of accuracy at a specific error budget. Angle Accuracy (AA) at the threshold *t* can be written in a general form as:

(18) $${\mathrm{AA}}_{M}\left(t\right)\;=\;\frac{1}{|U_{M}|}\sum_{u\in U_{M}}I\left[\;e(u)\leq t\;\right],$$

where $U_{M}$ is the set of evaluation units for metric *M* (e.g., ground-truth VPs for recall, predicted VPs for precision or images for image success rate), e(u) is the angular error associated with unit *u* under the chosen matching protocol, and I[·] is the indicator function.

In our experiments, recall AA, image success rate AA, and precision AA are computed over thresholds $t\in [0,10^{{}^{\circ}}]$, allowing us to characterize the performance of our pipeline from strict to relaxed angular tolerances.

## 5. Experimental Evaluation

### 5.1. Experimental Results

This section presents the results achieved by the proposed DRR-MMT-VPD algorithm and compares its performance with two state-of-the-art methods, J-Linkage [20] and CONSAC [23], on two vanishing point datasets. The computational performance of the proposed framework was evaluated by calculating the average processing time per image. It was noted that the average runtime per image is approximately 3 seconds without the MSAC-based arbiter stage, showing the performance efficiency of the base pipeline. When the arbiter stage is added, the runtime increases to approximately 6 s because of the additional refinement steps that enhance the accuracy and stability of the detected VPs. Although the additional refinement steps increase the computation time, they help enhance the VP selection, especially in noisy scenes. Nevertheless, runtime complexity can be optimized by incorporating performance optimization strategies like parallelization, vectorization, and image size standardization, in addition to GPU acceleration. In addition, the arbiter stage can be optionally disabled depending on the requirements of the application, allowing a trade-off between accuracy and performance. The proposed framework overall demonstrates potential to run in almost real time under optimized implementation.

#### 5.1.1. Performance on the Toulouse Vanishing Points Dataset (TVPD)

When applied over the TVPD, our method demonstrates the most reliable high-accuracy vanishing point detection, particularly at strict angular tolerances. Using Angle Accuracy (AA), as reported in Table 4, the highest recall is achieved at 1° and 3° (AA@1° = 0.827, AA@3° = 0.894). The highest image success rate is also achieved at the same thresholds (AA@1° = 0.578, AA@3° = 0.736), indicating that the predicted vanishing points are accurate and consistent at the image level. Although CONSAC reaches higher values at the loose 10° threshold (recall AA@10° = 0.95, image success rate AA@10° = 0.859), the AUC results, summarized in Table 5, confirm the stronger overall behavior of our method across thresholds up to the 10° cap; the best recall AUC is achieved at every tolerance (AUC@1° = 0.607, AUC@3° = 0.781, AUC@10° = 0.878), and the best image success rate AUC is likewise achieved across all tolerances (AUC@1° = 0.309, AUC@3° = 0.551, AUC@10° = 0.726).

**Figure 8:**
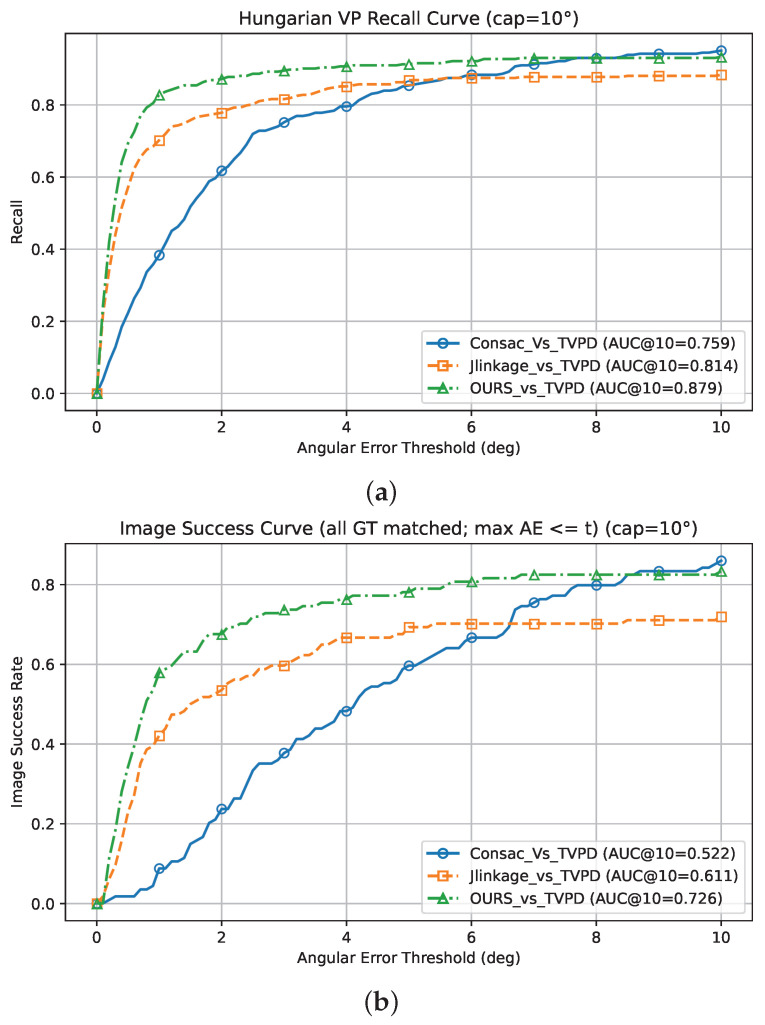
Recall and image success rate versus angular-error threshold (degrees) for vanishing point detection on the Toulouse dataset (TVPD), comparing *J-Linkage*, *CONSAC*, and *DRR_MMT_VPD*. The curves plot recall and the image success rate as functions of the tolerance θ; the legend reports AUC@$10^{{}^{\circ}}$, where higher values indicate better performance. (**a**) Recall AUC. (**b**) Image success rate AUC.

Figure 8 reports the Hungarian-matching recall and image success rate AUC curves as functions of the angular-error threshold, applied over the TVPD, with all AUC values computed under a 10° cap (AUC@10°).

#### 5.1.2. Performance on the York Urban Dataset (YUD)

On the YUD, our method delivers the strongest performance in the strict angular-error regime, which is the most indicative of geometric accuracy. In terms of recall (AA), as shown in Table 6, AA@1° = 0.819 is achieved, outperforming both CONSAC (0.673) and J-Linkage (0.710); this advantage is also reflected at the image level, where our image success rate reaches AA@1° = 0.59 and AA@3° = 0.80, compared to 0.26/0.74 for CONSAC and 0.43/0.68 for J-Linkage. Although CONSAC performance improves with the looser tolerance of 10° (recall 0.982, image success rate 0.95), our method remains competitive (recall 0.931, image success rate 0.84) and consistently exceeds J-Linkage at 3° and 10°. The AUC curves, summarized in Table 7, support these trends across thresholds; our method achieves the highest recall AUC at 1° and 3° (0.607 and 0.788) and the highest image success rate AUC across all caps (0.32, 0.595, 0.742), indicating a better overall ranking and precision–recall balance of the VP hypotheses at all error thresholds, especially below 3°.

**Figure 9:**
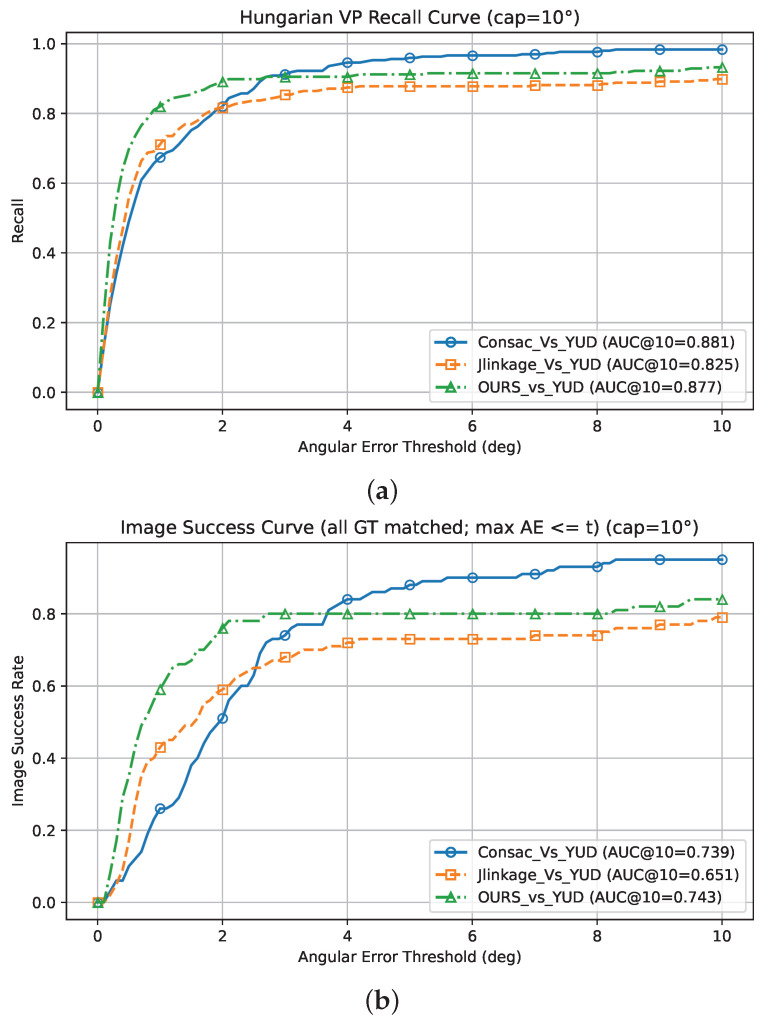
Recall (**a**) and image success rate (**b**) versus angular-error threshold in degrees for vanishing point detection on the York Urban Dataset (YUD), comparing *J-Linkage*, *CONSAC*, and *DRR_MMT_VPD*. The curves plot recall and the image success rate as functions of the tolerance θ; the legend reports AUC@$10^{{}^{\circ}}$, where higher values indicate better performance.

Figure 9 reports the Hungarian-matching recall and image success rate AUC curves as functions of the angular-error threshold, applied on the YUD, with all AUC values computed under a 10° cap (AUC@10°).

#### 5.1.3. Precision Under Multi-VP Prediction Across YUD and TVPD

Table 8 shows that our approach achieves the highest recall AUC@1° on both datasets (0.607 on the YUD and 0.607 on the TVPD), indicating improved recovery of ground-truth vanishing points under a strict 1° tolerance. Precision is slightly lower than J-Linkage—particularly on the TVPD (0.498 vs. 0.510)—which is expected because our pipeline often predicts up to four vanishing points (rather than three), increasing the number of hypotheses and, consequently, the likelihood of unmatched detections. Overall, these results highlight a clear trade-off: higher coverage (recall) at tight thresholds, with a modest reduction in precision due to the expanded VP output.

#### 5.1.4. Top-*N* VP Performance on the York Urban Dataset

**Figure 10:**
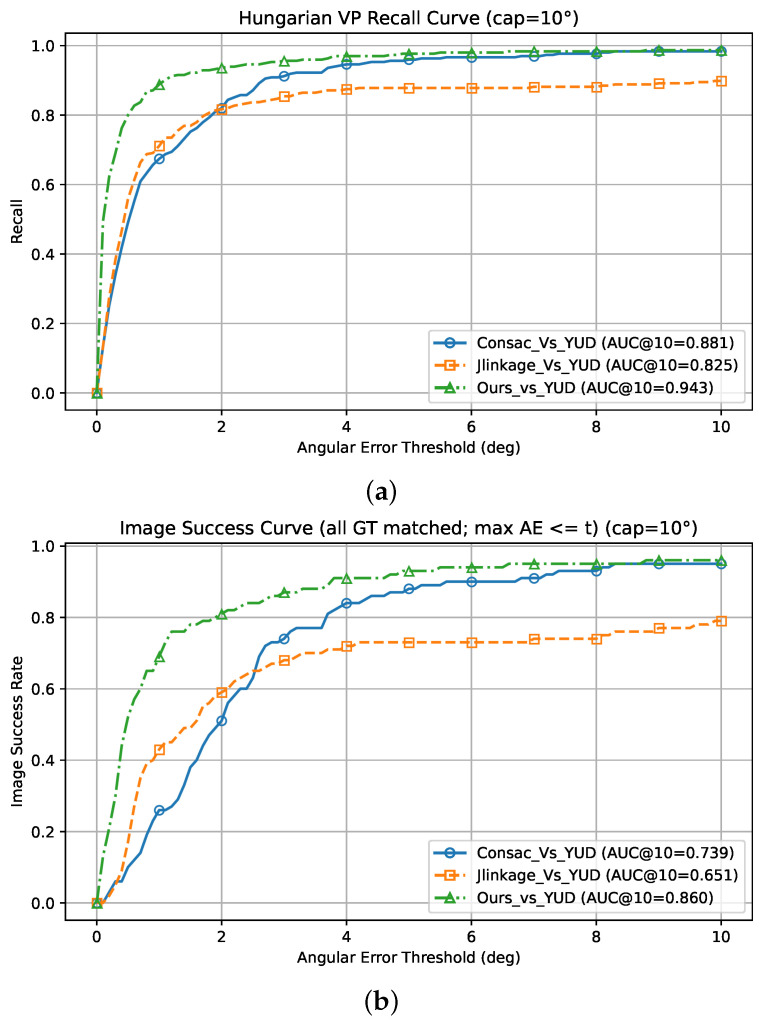
Recall (**a**) and image success rate (**b**) AUC plots on the York Urban Dataset (YUD) comparing state-of-the-art methods, CONSAC and J-Linkage, against our Top-*N* candidate pool. The high upper-bound performance shows that correct vanishing points are reliably generated within our candidate hypotheses.

In this study, the performance of the proposed DRR-MMT-VPD is evaluated by considering the Top-*N* vanishing point (VP) candidates generated prior to the final arbitration stage. By expanding the selection pool, it is shown that the correct vanishing points are consistently included among the predicted candidates, suggesting that the main performance limitation lies in the selection stage rather than in candidate generation. As shown in Table 9, the Top-*N* variant achieves significant gains over the YUD, reaching a recall of 0.887 at the strict 1° threshold, compared to 0.673 for CONSAC and 0.71 for J-Linkage. This high reliability at narrow tolerances indicates that our geometric trend partitioning effectively captures true VP locations. Furthermore, the Area Under the Curve (AUC) results in Table 10 confirm the importance of this trend partitioning technique across all thresholds, showing that our method achieves a recall AUC of 0.943 at a 10° cap, substantially outperforming state-of-the-art competitors. Based on the curves in Figure 10, it is obvious that our code provides a better pool of candidate hypotheses, and that for the vast majority of images, the correct set of VPs exists within our candidates, as illustrated specifically in the image success rate plot in Figure 10b. These results prove the effectiveness of our multi-trend discovery and suggest that while the arbiter may occasionally miss-detect the optimal candidate, the underlying detection framework is highly robust.

Overall, our method consistently attains the highest AUC@$\theta^{{}^{\circ}}$ across the full 1–$10^{{}^{\circ}}$ range, showing that it performs more reliably than the baselines as the angular-error tolerance is varied. For AA@$\theta^{{}^{\circ}}$ our method is the best in the narrow ranges 1–$3^{{}^{\circ}}$; however, CONSAC marginally outperforms in the wider ranges (≥5°), which is indicative of its bias in a recall toward loose criteria. What this means is that our detector is the most reliable in instances where strict geometric accuracy is of the utmost importance, while other methods may demonstrate better or similar performance when geometric accuracy is loosened.

#### 5.1.5. Discussion and Limitations

Considering all previous outcomes, it can be concluded that the suggested approach profits from having a distinct and modular geometric design. More specifically, the RANSAC stage, which is based on midpoints, efficiently captures the principal directions of the scene and reorganizes the detected line segments to form coherent trends prior to the vanishing point estimation. This type of behavior is particularly advantageous in urban environments that are structured because they are typically characterized by strong linear perspective cues. Moreover, the Top-*N* analysis proves that the correct vanishing points are almost all retained, indicating the strong efficacy of the multi-trend discovery stage. This means that, despite the arbiter not always choosing the best candidate, the detection pipeline is still effective. The rescue stage also increases reliability in challenging scenarios. The results also demonstrate that the method is sensitive to the quality of extracted line segments and to parameter tuning that may be mandatory when applied to different datasets. The proposed framework relies on straight-line geometry; hence, in scenes with strong curvature or limited geometric regularity, the performance of the framework is expected to be less stable.

### 5.2. Ablation Study

This section reports an ablation study of the proposed DRR-MMT-VPD pipeline for vanishing point (VP) detection on the YUD benchmark. This study isolates the contribution of:

(i) Trend fitting for separating dominant line families;
(ii) The arbiter used to select the best VP candidate;
(iii) A rescue mechanism that recovers missed VPs when the primary selection fails.

Evaluation uses a center-referenced angular error and one-to-one Hungarian matching to compute VP-level accuracy, recall/precision, and image-level success.

#### 5.2.1. Ablation Settings

Experiments are conducted on the YUD. The evaluation set contains 100 tested images with ground truth (images with missing sizes or absent predictions are excluded), totaling 294 ground-truth VPs. Four ablation settings are evaluated against the full pipeline (FULL):

- FULL: The complete proposed pipeline includes geometric trend partitioning (two horizontal trends and two vertical trends), the arbiter, and the rescue stage.
- A1_NO_TREND: Removal of the trend fitting module; all filtered line segments are pooled without geometric partitioning prior to the generation of the candidate VP.
- A2_ONE_TREND: The system forces a single trend per direction, simulating a single-VP detection approach.
- A3_NO_ARBITER: Disables the arbiter that resolves competing VP candidates per trend.
- A4_NO_RESCUE: Disables the rescue stage that attempts to recover a VP when the primary selection fails.

#### 5.2.2. Results and Discussion

Table 11 summarizes the ablation results of DRR-MMT-VPD on the YUD (100 images, 294 ground-truth VPs) and shows that geometric trend partitioning is the dominant contributor to reliable VP detection.

Starting from the reference configuration, the FULL pipeline provides the strongest overall trade-off, achieving the best combined performance in both image-level success and recall (success @3° = 0.72; success AUC = 0.685; recall AUC = 0.848). The relatively large number of predicted VPs in FULL (363) is expected, since the pipeline predicts one VP per trend and extracts two trends for each of the horizontal and vertical directions, which means around four predicted VPs per image, if all VPs are detected, compared to three ground-truth VPs per image, which can reduce precision. For scoring the best results, the FULL setting is used as the reference configuration.

In contrast, A1_NO_TREND shows that deactivating the trend extraction stage causes the pipeline to degenerate into pooled candidate generation, considerably reducing completeness and producing a very low image success rate at 3° (success @3° 0.72→0.04; success AUC 0.685→0.039; recall AUC 0.848→0.596). This behavior implies a frequent failure to jointly recover all ground-truth VPs within the same image.

Concerning A2_ONE_TREND, it shows a degradation that is less pronounced but still considerable. Enforcing a single trend performs better than canceling trend fitting entirely, but it remains significantly inferior to FULL (success @3° remains 0.04; recall @3° 0.605→0.622; success AUC remains 0.039; recall AUC 0.596→0.605). This result confirms that separating multiple dominant line families is advantageous, even within the same directional group. Analyzed together, A1 and A2 demonstrate that the image success rate collapses to 0.04 when trend-based discovery is either removed or overly restricted, showing that the system cannot effectively resolve the complex orthogonal structure of Manhattan scenes without explicit trend separation. Although these settings imply a relatively high precision, the result is misleading and primarily reflects weak VP detection, as proved by the substantial drop in recall AUC to approximately 0.60.

A3_NO_ARBITER presents a different pattern. Disabling the arbiter produces the highest Median AE (0.382 degrees), lowers precision at 3°, and reduces both the recall AUC and image success rate AUC relative to FULL (success AUC 0.685→0.636; recall AUC 0.848→0.825), while maintaining the total number of predicted VPs nearly unchanged. This indicates that the arbiter mainly improves candidate selection quality rather than candidate generation itself.

Finally, A4_NO_RESCUE yields results that remain close to those of FULL, indicating that the rescue module primarily benefits a limited subset of difficult images, whereas the combination of trend extraction and arbitration accounts for most of the overall performance.

In general, the FULL pipeline achieves the highest success AUC (0.685) and recall AUC (0.848). These results support the multi-trend design of DRR-MMT-VPD and define the arbiter as a key component for VP selection stability, while the rescue mechanism serves as a complementary safeguard for challenging cases.

## 6. Conclusions and Future Work

This paper introduces DRR-MMT-VPD as a training-free framework, which is transparent and interpretable, and meant to be a reliable and accurate vanishing points (VPs) detector. By integrating midpoint–orientation representation, dual consensus mechanisms, and density-based clustering, the system achieves stable VP localization without requiring supervision or Manhattan-world assumptions.

This dual RANSAC pipeline integrates regression in the midpoint–orientation space, in addition to an MSAC-based selector, which preserves balance between reliability and accuracy.

The experimental results we applied to the YUD and TVPD benchmarks demonstrate that the DRR-MMT-VPD approach achieves higher accuracy than J-Linkage and CONSAC across various angular thresholds, preserving competitive and reliable performance in complex environments.

For future work, we plan to upgrade pipeline performance by improving the arbiter selection strategy, avoiding hard manual tuning by introducing an efficient automatic parameter adaptation strategy, and decreasing processing time while conserving efficiency and granting reliable performance in real-time scenes. This will allow the pipeline to operate consistently across a wider range of scenes while preserving its interpretable nature. Additionally, we plan to explore hybrid variants that integrate lightweight learned priors into the consensus framework, combined with computational optimizations, in order to enhance real-time operation and mainly to enable the application of DRR-MMT-VPD to downstream tasks like camera calibration and 3D reconstruction, where accurate VP estimation provides a strong geometric basis for higher-level vision.

## Figures and Tables

**Table 1:** Comparison of representative vanishing point detection techniques and the proposed DRR-MMT-VPD framework.

Representative Techniques	Method Family	Main Weaknesses	Main Strengths
J-Linkage [20]	Multi-model clustering	Computational cost at scale	Model-number agnostic
T-Linkage [16]		Sampling parameter sensitivity	Multi-structure recovery
J-Linkage [20]		Weak-separation ambiguity	Outlier tolerance
Direct intersections [22]	Geometric	Strong scene priors	Geometric interpretability
Manhattan-world [17]		Sensitivity to line errors	Efficient inference
Atlanta-world [9]		Limited generalization	Strong structured-scene performance
DeepVP [11] CONSAC [23] NeurVPS [24] Geometry-aware deep models [25] Transformer-based methods [26,27]	Learning-based Learning-guided	Training-data dependence Domain-shift sensitivity Higher computational cost	Global-context reasoning Learned geometric priors Strong benchmark accuracy
DRR-MMT-VPD (Ours)	Geometric Consensus-based Clustering-assisted	Line-quality dependence Threshold sensitivity Stagewise error propagation	Training-free Midpoint-based multi-trend RANSAC Early directional separation Rescue-based VP recovery

**Table 2:** Main preprocessing and filtering operations used in Section 3.1.

Stage	Operation	Parameter(s)	Purpose/Effect
Contrast enhancement	CLAHE [28]	Contrast flag; clip limit; tile grid size	Enhances local contrast.
Optional binarization	Adaptive thresholding [29]	Adaptive threshold flag	Supports uneven illumination handling.
Edge-preserving smoothing	Bilateral filtering [30]	*d*; $\sigma_{\mathrm{color}}$; $\sigma_{\mathrm{space}}$	Smooths noise while preserving edges.
Noise suppression	Gaussian filtering [31]	Kernel size; $\sigma_{g}$	Reduces residual high-frequency noise.
Line extraction	LSD [5] and length filtering	Refinement mode; $L_{\min}$	Extracts line segments and removes short ones.
Directional filtering	Horizontal or vertical selection	Angular threshold	Separates segments by direction.
Geometric normalization	Endpoint ordering and angle normalization	Ordering rule; angle interval	Standardizes segment representation.
Branch separation	Horizontal and vertical processing	Retained directional subsets	Processes each directional family independently.

**Table 3:** Concise comparison of the York Urban Dataset and the Toulouse Vanishing Points Dataset.

Characteristic	York Urban Dataset	Toulouse Vanishing Points Dataset
Scene type	Urban scenes	Manhattan-like scenes
Number of images	102	114
Ground truth	Three orthogonal vanishing directions	Three orthogonal vanishing directions
Annotation format	Point-based ground truth	Ground truth supported by synchronized inertial data with uncertainty regions

**Table 4:** Angle Accuracy (AA) evaluation on the TVPD. Recall and image success rate are reported at angular-error thresholds of 1°, 3° and 10° (AA@t). Best results per column are highlighted in bold.

	Recall			Image Success Rate		
Method	AA@1°	AA@3°	AA@10°	AA@1°	AA@3°	AA@10°
CONSAC	0.383	0.751	**0.95**	0.087	0.377	**0.859**
J-Linkage	0.701	0.815	0.883	0.421	0.596	0.719
Ours	**0.827**	**0.894**	0.932	**0.578**	**0.736**	0.833

**Table 5:** Area Under the Curve (AUC) summary on the TVPD. AUC values for recall and image success rate are reported at angular-error thresholds of 1°, 3°, and 10° (AUC@t). Best results per column are highlighted in bold.

	Recall			Image Success Rate		
Method	AUC@1°	AUC@3°	AUC@10°	AUC@1°	AUC@3°	AUC@10°
CONSAC	0.211	0.472	0.758	0.023	0.16	0.521
J-Linkage	0.51	0.688	0.813	0.218	0.427	0.61
Ours	**0.607**	**0.781**	**0.878**	**0.309**	**0.551**	**0.726**

**Table 6:** Angle Accuracy (AA) evaluation on the YUD. Recall and image success rate are reported at angular-error thresholds of 1°, 3°, and 10° (AA@t). Best results per column are highlighted in bold.

	Recall			Image Success Rate		
Method	AA@1°	AA@3°	AA@10°	AA@1°	AA@3°	AA@10°
CONSAC	0.673	**0.911**	**0.982**	0.26	0.74	**0.95**
J-Linkage	0.71	0.853	0.897	0.43	0.68	0.79
Ours	**0.819**	0.904	0.931	**0.59**	**0.8**	0.84

**Table 7:** Area Under the Curve (AUC) summary on the YUD. AUC values for recall and image success rate are reported at angular-error thresholds of 1°, 3°, and 10° (AUC@t). Best results per column are highlighted in bold.

	Recall			Image Success Rate		
Method	AUC@1°	AUC@3°	AUC@10°	AUC@1°	AUC@3°	AUC@10°
CONSAC	0.441	0.687	**0.881**	0.104	0.374	0.738
J-Linkage	0.484	0.696	0.824	0.194	0.447	0.65
Ours	**0.607**	**0.788**	0.876	**0.32**	**0.595**	**0.742**

**Table 8:** AUC@$1^{{}^{\circ}}$ comparison on YUD and TVPD (recall and precision). Best results per column are highlighted in bold.

	YUD		TVPD	
Method	Recall (AUC@1°)	Precision (AUC@1°)	Recall (AUC@1°)	Precision (AUC@1°)
Consac_Vs_YUD	0.441	0.185	0.211	0.09
Jlinkage_Vs_YUD	0.484	0.474	0.51	**0.51**
OURS_Vs_YUD	**0.607**	**0.479**	**0.607**	0.498

**Table 9:** Angle Accuracy (AA) evaluation with Top-*N* VPs on the YUD. Recall and image success rate are reported at angular-error thresholds of 1°, 3° and 10° (AA@ θ). Best results are highlighted in bold.

	Recall			Image Success Rate		
Method	AA@1°	AA@3°	AA@10°	AA@1°	AA@3°	AA@10°
CONSAC	0.673	0.911	0.982	0.26	0.74	0.95
J-Linkage	0.710	0.853	0.897	0.43	0.68	0.79
Ours (Top-*N*)	**0.887**	**0.955**	**0.986**	**0.69**	**0.87**	**0.96**

**Table 10:** Area Under the Curve (AUC) summary for the Top-*N* study on the YUD. Best results per column are highlighted in bold.

	Recall			Image Success Rate		
Method	AUC@1°	AUC@3°	AUC@10°	AUC@1°	AUC@3°	AUC@10°
CONSAC	0.441	0.687	0.881	0.104	0.374	0.738
**J-Linkage**	0.484	0.696	0.824	0.194	0.447	0.650
Ours (Top-*N*)	**0.728**	**0.863**	**0.943**	**0.438**	**0.683**	**0.859**

**Table 11:** Ablation metrics on the YUD (100 images, 294 ground-truth VPs), summarizing the comparative results for the different system configurations. Best results per column are highlighted in bold.

Ablation Case	Pred. VPs	Matched Pairs	Median AE (deg)	Img Succ @ 3°	Recall @ 3°	Prec. @ 3°	Succ AUC (0–10°)	Recall AUC
FULL	363	**293**	0.322	**0.720**	**0.874**	0.708	**0.685**	**0.848**
A1_NO_TREND	200	200	0.300	0.040	0.605	0.890	0.039	0.596
A2_ONE_TREND	200	200	**0.251**	0.040	0.622	**0.915**	0.039	0.605
A3_NO_ARBITER	363	**293**	0.382	0.660	0.847	0.686	0.636	0.825
A4_NO_RESCUE	**364**	**293**	0.319	**0.720**	**0.874**	0.706	0.677	0.847

## Data Availability

Dataset available on request from the authors.

## Abbreviations

AA	Angle Accuracy
AE	Angular Error
AUC	Area Under the Curve
CLAHE	Contrast-Limited Adaptive Histogram Equalization
CONSAC	Conditional Sample Consensus
DBSCAN	Density-Based Spatial Clustering of Applications with Noise
DRR-MMT-VPD	Dual RANSAC with Rescue Midpoint-based Multi-Trend Vanishing Point Detection
LSD	Line Segment Detector
MAD	Median Absolute Deviation
MLESAC	Maximum Likelihood Estimation Sample Consensus
MSAC	M-Estimator Sample Consensus
PROSAC	Progressive Sample Consensus
RANSAC	Random Sample Consensus
TVPD	Toulouse Vanishing Points Dataset
VP	Vanishing Point
YUD	York Urban Dataset
